# 3d elastic-modulus imaging using ultrasound linear arrays and efficient data-driven training strategies

**DOI:** 10.1007/s10237-026-02056-8

**Published:** 2026-03-30

**Authors:** Will Newman, Jamshid Ghaboussi, Michael F. Insana

**Affiliations:** 1 Grainger College of Engineering, Department of Bioengineering, University of Illinois at Urbana-Champaign, Urbana, IL 61801 USA; 2 Beckman Institute of Advanced Science and Technology, Urbana, IL 61801 USA; 3 Grainger College of Engineering, Department of Civil and Environmental Engineering, University of Illinois at Urbana-Champaign, Urbana, IL 61801 USA

**Keywords:** Constitutive modeling, Elastography, Finite-element analysis, Machine learning, Ultrasound

## Abstract

We are developing ultrasonic-based techniques for elastic modulus imaging throughout a tissue volume using the autoprogressive (AutoP) method with linear array transducers. AutoP discovers the constitutive properties of media by estimating the stress and strain in a volume from force-displacement measurements recorded in planes. AutoP is a data-driven machine learning technique that combines shallow neural network structures with object-specific measurements, in stark contrast with traditional deep learning techniques. In this paper, we outline a strategy for acquiring measurements from a series of compression planes across the volume and applying them in a training sequence that efficiently trains networks to model volumetric deformation patterns. We show with phantom studies that measurements collected while compressing the medium in parallel planes throughout the volume can yield elastic modulus image values within 10% of values measured independently. Deformation models are developed accurately in minutes using a comprehensive set of exact measurements. However, experimental limitations slow the learning process and ultimately limit the contrast and spatial resolution of modulus images in ways that can be minimized. An efficient imaging strategy balances the need to provide more planes of force-displacement measurements to enhance learning with the need to manage measurement errors. Test statistics obtained from the developing model can guide the learning process.

## Introduction

As mechanobiologists work to decipher complex relationships between abnormal cell behavior and the changing mechanical environment of the tissue (Xin et al. 2023), image scientists are striving to map the mechanical properties of the tissues that surround and nourish cells (Dietrich et al. 2024; Mariappan et al. 2010). The link between these two fields could offer new tools for studying the role of mechanobiology in sustaining health and suppressing disease. Quantitative 3-D elasticity imaging with millimeter-scale resolution is a step toward discovering which mechanical features of tissues prompt cellular responses, in vivo (Saraswathibhatla et al. 2023).

Ultrasonic elastography (Ophir et al. 1991) is an approach similar to manual palpation that replaces fingertip sensing with a linear array transducer. The resulting images vary with the applied load and the technique for monitoring deformations (Sarvazyan et al. 1995; Doyley and Parker 2014). We employ quasi-static loading methods that allow time for the applied force to propagate throughout the entire contiguous medium. Simultaneously, internal displacements resulting from the applied load are measured in the ultrasonic scan plane using a robust speckle tracking algorithm (Hashemi and Rivaz 2017).

The core analysis problem that is addressed is the extraction of constitutive properties in a deformed volume from force-displacement measurements. Constitutive properties include the materials and structures within a volume that determine the stress and strain patterns that develop when the medium is loaded. A common assumption with strain imaging using quasi-static compression is that the deformed material is constrained to a plane stress geometry with constant in-plane stress. Under these assumptions, the strain image contrast corresponds to the variations in material properties (Barbone and Oberai 2007). However, controlling heterogeneous tissue movements is unrealistic in practice, so the stress field must also be measured and combined with the strain field to avoid artifacts. Constitutive relationships are difficult to measure accurately without fully accounting for stress and strain in the deformed volume. For this reason, clinical applications of elastography require physicians to assess tissue properties by interpreting qualitative, contrast-based planar images to separate tissue structure from artifacts. This is true for quasi-static (Cui et al. 2022) and dynamic (Gennisson et al. 2013) loading techniques. Despite limitations, imaging of qualitative elasticity has shown diagnostic promise (Cho et al. 2012).

The autoprogressive (AutoP) method (Ghaboussi et al. 1998) can describe the constitutive behavior of deformed media because it estimates all components of stress and strain from a limited sampling of force and displacement measurements (Hoerig et al. 2021). AutoP takes a data-driven approach to solving the constitutive inverse problem by propagating force-displacement measurements through finite-element analysis (FEA) to generate physically constrained stress–strain training data. Those data inform the training of a Cartesian neural network constitutive model (CaNNCM) (Hoerig et al. 2018) from which elastic modulus images are formed. Learned solutions are not unique, but they are consistent with the available data and, as such, the CaNNCM closely represents constitutive properties of the medium. The challenge is to identify the smallest set of measurements that can achieve acceptable image quality for a given task.

Imaging volumetric elasticity with an ultrasound linear array transducer requires the acquisition of several compression planes of force-displacement measurements that span the volume (Hoerig et al. 2020; Newman et al. 2024b). Blindly adding compression plane data increases the computation time but may not improve planar image quality (Newman et al. 2024b). The value to model building contributed by data from a compression plane must exceed the degradation caused by the inevitable measurement errors (Newman et al. 2024a). Selecting compression planes and scheduling when to introduce them during training must be carefully supervised to achieve high-quality volumetric images in the least amount of training time. This report summarizes a series of experiments designed to develop training strategies for volumetric elasticity imaging using ultrasound linear arrays.

Although this paper describes methods for imaging volumetric elasticity, it also documents a search for accessible measurement information that efficiently informs the construction of constitutive models using machine learning techniques.

## Methods

Details regarding the mathematical framework for AutoP training, network architectures, and training strategies can be found in Hoerig et al. (2020, 2021); Newman et al. (2024b). This section provides a brief overview of AutoP, describing how planar and volumetric elastic modulus images are formed from planar force-displacement measurements.

**Fig. 1:**
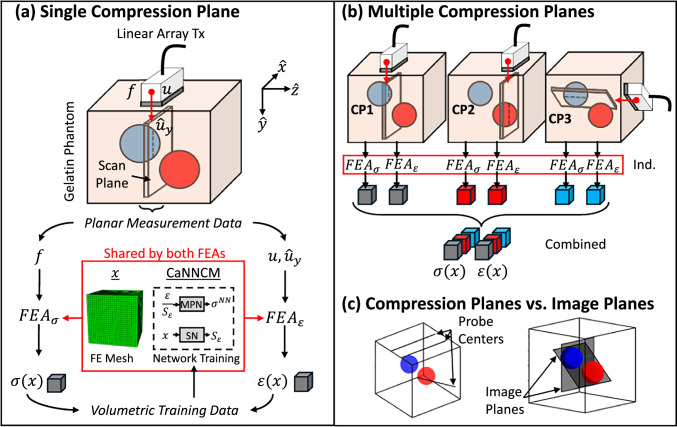
**a** Measurement data are acquired by compressing a linear array ultrasound probe into a cubic gelatin phantom while recording the applied surface force *f*, probe displacement *u*, and echo frames used to estimate the internal *y*-component of displacement $\hat{u}_y$. AutoP uses two FEAs per compression plane to convert planar measurements into a set of volumetric stress–strain training data that is applied in the development of a CaNNCM. **b** Volumetric modeling involves applying multiple compression planes that are combined during CaNNCM training. **c** The image planes may be independent of the compression planes

### The autoprogressive method

The autoprogressive method (AutoP) for ultrasonic elasticity imaging begins with measurements of force and displacements in one or more compression planes. A linear array ultrasound probe is slowly pressed into the tissue a few millimeters while measuring the applied surface force *f*, probe displacement *u*, and axial displacements $\hat{u}_y$ describing the internal tissue deformation in the plane.

The 3D surface geometry of the object and compressor are modeled in CAD software, and the undeformed sample volume is meshed as shown in Fig. 1a. That meshed volume is connected to two copies of finite-element analysis (FEA) software, where the measured forces are applied as a boundary condition on the compressor in $\textrm{FEA}_\sigma$ and the measured displacements are applied as boundary conditions in $\textrm{FEA}_\varepsilon$. Stresses $\sigma (x)$ are computed from force measurements in $\textrm{FEA}_\sigma$ and strains $\varepsilon (x)$ from displacement measurements in $\textrm{FEA}_\varepsilon$ at mesh integration points *x* in the volume. FEAs physically constrain the solution space for stress and strain as neural networks learn material properties.

Figure 1a illustrates the process of converting force and displacement measurements from one compression plane into volumetric stress and strain training data. The gray cubes represent sets of training data from the entire volume that emerge from the two FEAs.

Two neural networks are tasked with learning different aspects of volumetric stress and strain as measurement data flow through the FEAs. The material property network (MPN) is a feed-forward, multi-layered neural network that receives $\varepsilon (x)$ from $\textrm{FEA}_\varepsilon$ and learns stresses $\sigma ^{NN}(x)$ from comparisons with $\sigma (x)$ values computed by $\textrm{FEA}_\sigma$. The two stress fields very quickly converge to a lax criterion as MPN network weights are adjusted. No positional information is encoded in the MPN because its goal is to learn spatially averaged mechanical properties throughout the deformed volume.

Residual differences between $\sigma ^{NN}(x)$ and $\sigma (x)$ arise at locations in the volume where the material properties deviate from the spatial average. To adjust for these differences, strain scale factors $S_\varepsilon$ are estimated at each mesh location *x* by a separate neural network – the spatial network (SN) – and are used to scale the strain input to the MPN. Scale factors are adjusted to model the spatially varying deformations that eventually become image contrast in the elastic modulus image. As the model converges to an increasingly stringent criterion, the deformation model improves so that elastic modulus image contrast increases in a quantitatively accurate manner (Hoerig et al. 2018; Newman et al. 2024b).

During network training, no reference is made to a mathematical constitutive model. After model convergence, the network weights in the MPN and SN encode a nonparametric mapping of the spatially varying constitutive behavior of the deformed volume. Together, the MPN and the SN compose a Cartesian neural network constitutive model (CaNNCM) that is later probed to estimate image parameters. The CaNNCM replaces the constitutive model normally used to form the stiffness matrix used in standard FEA software (Hashash et al. 2004). In Abaqus finite-element software (Abaqus Software, Dassault Systèmes SE, Vélizy-Villacoublay, France), a user-defined material is created to define nonparametric, local material stiffness (Jacobian) matrices at each integration point based on the current state of the CaNNCM. Local matrices are used to form the global stiffness matrix in the FEA equations, which considers material properties, element connectivity, and boundary conditions of the entire continuum.

### Training cycles

Displacement measurements input to $\textrm{FEA}_\varepsilon$ describe the deformation pattern in the compression plane, which is influenced by spatial variations in material properties throughout the deformed volume, the shape and magnitude of the applied load, and external boundary conditions. The force sensed at the probe surface that is entered into $\textrm{FEA}_\sigma$ describes net properties of the medium, but provides no spatial information. The two FEAs work together to model the spatial distribution of material properties by adjusting the CaNNCM until the two stress fields agree at points throughout the volume. In this way, the stresses and strains in the compressed volume are estimated without assuming a parametric constitutive model. The process of training with measurement data from one or many compression planes is diagrammed in Figs. 1a and b, respectively.

Each AutoP training cycle generates volumetric stress–strain training data from one or more sets of force-displacement measurements, exposes the networks to the training data, and updates the CaNNCM for use in the next FEA iteration (illustrated for one set of measurement data in the bottom of Fig. 1a). One AutoP cycle is executed for each load step in the sample volume compression. A pass through the measurement data refers to cycling through all recorded load steps.

Achieving a stringent network convergence criterion requires multiple passes through the measurements. We found (Newman et al. 2024b) that convergence is accelerated by training the MPN during the first pass while setting all scale factors from the SN to 1. In this way, the CaNNCM first learns spatially averaged properties. After the first pass, the MPN is fixed and only the SN is trained to estimate scale factors that account for spatial variations in properties about the mean. Scale factors adjust the slope of the average stress versus strain curve at each spatial location. Average properties are quickly learned by the MPN, while achieving SN convergence consumes a majority of the training time.

Network training takes place at all points in the contiguous volume, even for planar imaging. While the most accurate modulus image data are obtained within 2–3 mm of a compression plane where displacements are estimated (Newman et al. 2024b), the entire volume responds to each compression to affect all measurements. Despite only having measurement data from the compression plane(s), the model is also informed by the known initial boundary conditions (those in the undeformed state).

Multiple compression planes are recorded when the goal is to form images in arbitrary planes within the volume. Figure 1b indicates three compression plane experiments acquired over the phantom volume. Applying all three sets of measurement data within an AutoP cycle requires 6 FEA executions (2 per plane) yielding three volume sets of paired stress–strain training data. The combination constitutes a full set of available training data. The location and number of compression planes used in training are indicated in the Results Section by lines on the surface of the cubic phantom marking the center probe position (Fig. 1c). Image planes can be selected arbitrarily throughout the sampled volume as illustrated in Fig. 1c.

### Efficient training strategies for volumetric imaging

We sensed the volume using parallel compression planes separated by a distance determined by the elevational ultrasonic pulse width. For an 8 MHz linear array, a separation of at least 4 mm between compression planes was applied to sense a cubic volume of 32.8 $\textrm{cm}^3$. Developing volumetric deformation models from multiple compression planes is a time intensive process. The overall computational load is mainly determined by (a) the number of compression planes used within each AutoP cycle and (b) the density of the FE mesh. The number of compression planes determines the number of FEA executions within each AutoP cycle (see Fig. 1b). The mesh density controls the number of integration points where the stress–strain training data are expressed within the meshed volume. The computational load was reduced by sequentially introducing compression planes individually before training with all compression planes simultaneously during the last few passes.

#### Training schedule

The MPN was trained for one pass using the data from one compression plane to quickly learn the spatially averaged material properties. Then the SN was trained with the data from each compression plane individually for two passes. As AutoP iterates through each compression plane, the developing CaNNCM retains the properties learned from previous compression planes by maintaining a ‘window’ of training data history, where data from previously introduced planes are mixed for a time (Wang et al. 2022). Once all planes have been introduced individually, the CaNNCM then trained on the data from all compression planes simultaneously for 4 passes. In simulation, we have shown that this dynamic, multi-plane training schedule is a time-efficient way to develop accurate deformation models (Newman et al. 2024b).

The main finite-element mesh considered in this study consists of hexahedral mesh elements (C3D8, full-integration) with an average element length of 2-mm. This mesh is used when the adjacent compression planes are parallel (see CP1 and CP2 in Fig. 1b). For arbitrarily oriented compression planes (see CP3 Fig. 1b), we used 2-mm quadratic tetrahedral mesh elements (C3D10, full-integration) generated with the public-domain meshing software *gmsh* (Geuzaine and Remacle 2020).

#### Focusing training

While the measurement data enters the analysis at mesh nodes within the compression planes, each execution of FEA forms a set of training data at integration points throughout the contiguous volume. To focus network training on regions closest to the measurement data, we spatially subsample (Newman et al. 2024b) the training data points selected to participate in training for multi-plane models. In regions nearest the compression planes, where displacements are measured, all training data are included. Outside of these regions, only 10% of the training data samples are randomly selected for inclusion. Additionally, we spatially weight the update to the scale factors (Newman et al. 2024b) in a way that emphasizes the selected training data points closest to the displacement measurements. Spatial subsampling discards training data used in a training cycle, whereas weighting retains but adjusts the influence of all remaining training data. Both methods aim to place emphasis on regions near the measurements while maintaining a sense of the entire volume and minimizing training time.

### Test objects

**Fig. 2:**
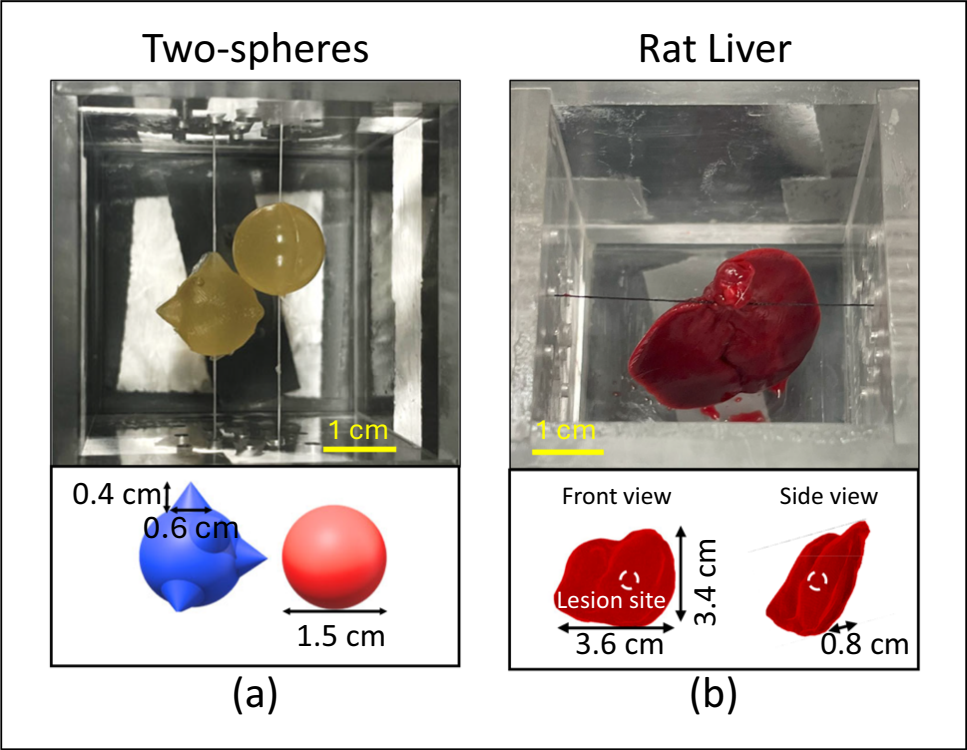
Inclusions are suspended within the phantom mold prior to being embedded in a background gel. Sutures were removed after the background gelatin congealed

Two cubic elasticity imaging phantoms were manufactured using a procedure reported previously (Hall et al. 1997). Both 50 mm gelatin cubes were cast from acrylic molds. Stiff gelatin inclusions were cast from 3-D printed molds. Hydrogel stiffness is controlled by the gelatin concentration. Embedded inclusions were positioned at known locations as shown in Fig. 2a. Young’s modulus *E* was measured for each gelatin phantom component using compression tests applied to separate cylindrical test samples as described previously (Wang et al. 2022).

The two-sphere phantom contained two gelatin inclusions (7.5 mm radius, *E* = 19.8 ± 0.7 kPa) embedded in a homogeneous background gel ($E = 7.9 \pm 0.4$ kPa). The sphere centers were offset from each other, so a compression plane could encounter one or both inclusions. One of the spheres was cast with five small cones, creating the rough surface diagrammed in Fig. 2a.

A different phantom used fresh *ex vivo* rat liver from a healthy adult male rat as the embedded inclusion in a gelatin background material. The right lobe of the liver tissue was first injected with 20 $\mu$L of formalin solution to induce a symmetric stiff lesion of diameter less than 8 mm ($E = 14.3 \pm 5.6$ kPa) in otherwise homogeneous tissue ($E = 4.7 \pm 1.3$ kPa). The use of biological tissues required refrigeration for storage, which accelerates the congealing process of gelatin. Consequently, the mean background gelatin material ($E = 17.4 \pm 1.4$ kPa) was stiffer than the liver tissue and stiff lesion. Compression planes were acquired after warming the liver phantom at room temperature for at least one hour. Afterward, the liver phantom was disassembled and cut into cubes for materials testing on relatively homogeneous sub volumes.

Key differences between the two phantoms are the inclusion shapes, size and material contrast. The two-sphere phantom has a homogeneous background surrounding fairly simple inclusions, only 2.5-times stiffer than the background material. The inclusion stiffnesses and geometries for this phantom were known with small uncertainties. For this reason, the majority of the results discussed in this report are from the two-sphere phantom. The rat liver phantom includes an irregularly-shaped biological tissue, which occupies a 3-times larger portion of the phantom volume than the inclusions in the two-sphere phantom. The untreated liver tissue was 3.7-times softer than the background gelatin and 3.0-times softer than the formalin-induced lesion. The dynamic range of material properties is larger in the rat liver phantom than in the two-sphere phantom. Our objective was to challenge AutoP using a complex variation of material properties throughout the 3D volume.

### Data acquisition and displacement estimation

The cubic phantoms were placed on a rigid nonslip surface and compressed from above in three load steps, 0.5 mm per load step, by a precisely positioned 8-MHz linear array ultrasound transducer (Siemens Sonoline Antares, Siemens Healthcare USA, Mountainview, CA). Only the transducer footprint was used to apply the displacement load. A force sensor (ATI Industrial Automation, Apex, NC) recorded the load that was applied by the 11 mm $\times$ 46 mm contact area of probe.

An RF echo frame was recorded after each compression step for displacement estimation. Echo data were sampled at 40 MHz by a linear array with a 0.2 mm transducer pitch. We used the GLobal Ultrasound Elastography (GLUE) algorithm developed by the Rivaz lab (Hashemi and Rivaz 2017) to track the interframe echo motion describing displacements in the scan plane. GLUE uses a smoothness constraint that estimates displacement at a point using information from the entire RF echo frame. While smooth displacements are ideal for our application, this constraint can impose a smooth bias rather than additive noise in displacement when the algorithm cannot accurately track the scatterer motion.

### Monitoring training progress and assessing results

One of our main objectives is to determine how to monitor model development so that we may intervene with additional measurements when necessary to achieve *E* images of acceptable quality. We typically do not have access to the underlying tissue material properties, but we can measure properties of the updating training data and the evolving elastic modulus image contrast as AutoP iterates through each pass. We developed three test statistics that focus on the evolution of the stress–strain training data (Newman et al. 2024b).

The three test statistics are: stress entropy $H(\sigma )$ (bits), strain reversibility $\eta _{\varepsilon }$, and stress convergence $\Delta \bar{\sigma }$ (kPa). They are measured using the training data determined after each pass. Together the three metrics indicate to an operator how much of the stress entropy is learned by the CaNNCM, the consistency of the training data formed by $\textrm{FEA}_{\sigma }$ and $\textrm{FEA}_{\varepsilon }$, and the point at which the CaNNCM finishes updating. $H(\sigma )$ and $\eta _{\varepsilon }$ use an information theoretic approach that characterizes properties of the stress–strain data probabilistically, whereas $\Delta \bar{\sigma }$ estimates the spatially averaged change in stress data between consecutive passes. They address the questions (a) has the last pass through the measurement data further enriched the training data, (b) has that additional information been successfully transferred into the CaNNCM, and (c) have the networks converged indicating additional data are required for training or training is complete.

$H(\sigma )$ measures the overall entropy in the six components of stress from $\textrm{FEA}_\sigma$ as calculated from the training data, weighting each component equally. As contrast in material properties emerges and $H(\sigma )$ increases, we look to $\eta _{\varepsilon }$ to monitor if that information is influencing the developing model. $\eta _{\varepsilon }$ is a measure of the mutual information between the evolving strains from $\textrm{FEA}_\sigma$, where only measured surface force is applied, and the strains from $\textrm{FEA}_\varepsilon$, where measured displacements are applied. The strains from $\textrm{FEA}_\varepsilon$ remain constant throughout training since only measured internal displacements are applied. An $\eta _{\varepsilon }=1.0$ means there is perfect agreement between the distributions of the two strain fields; that the strain field in $\textrm{FEA}_\sigma$ developed from force measurements and the CaNNCM is a close statistical match to the strain field computed from displacements in $\textrm{FEA}_\varepsilon$. We found previously that if the CaNNCM converges and $\eta _{\varepsilon }\le 0.7$, then we conclude that the CaNNCM was not able to obtain agreement between the two strain fields, suggesting additional measurement data could be needed. The CaNNCM has finished updating once the curves of strain reversibility and stress entropy versus pass number plateau and $\Delta \bar{\sigma }\longrightarrow 0$. These metrics are intended to be used together to understand how the influence of new measurement data can be assessed from changes in these estimates.

## Results

A series of planar and volumetric phantom imaging studies were conducted to demonstrate procedures and compare strengths and weaknesses. Planar imaging refers to the application of a single compression plane of measurement data to form an image coincident to that plane (see Fig. 1a). Volumetric imaging refers to the application of many compression planes of measurement data to form a 2-D image in an arbitrary plane (see Fig. 1b).

**Fig. 3:**
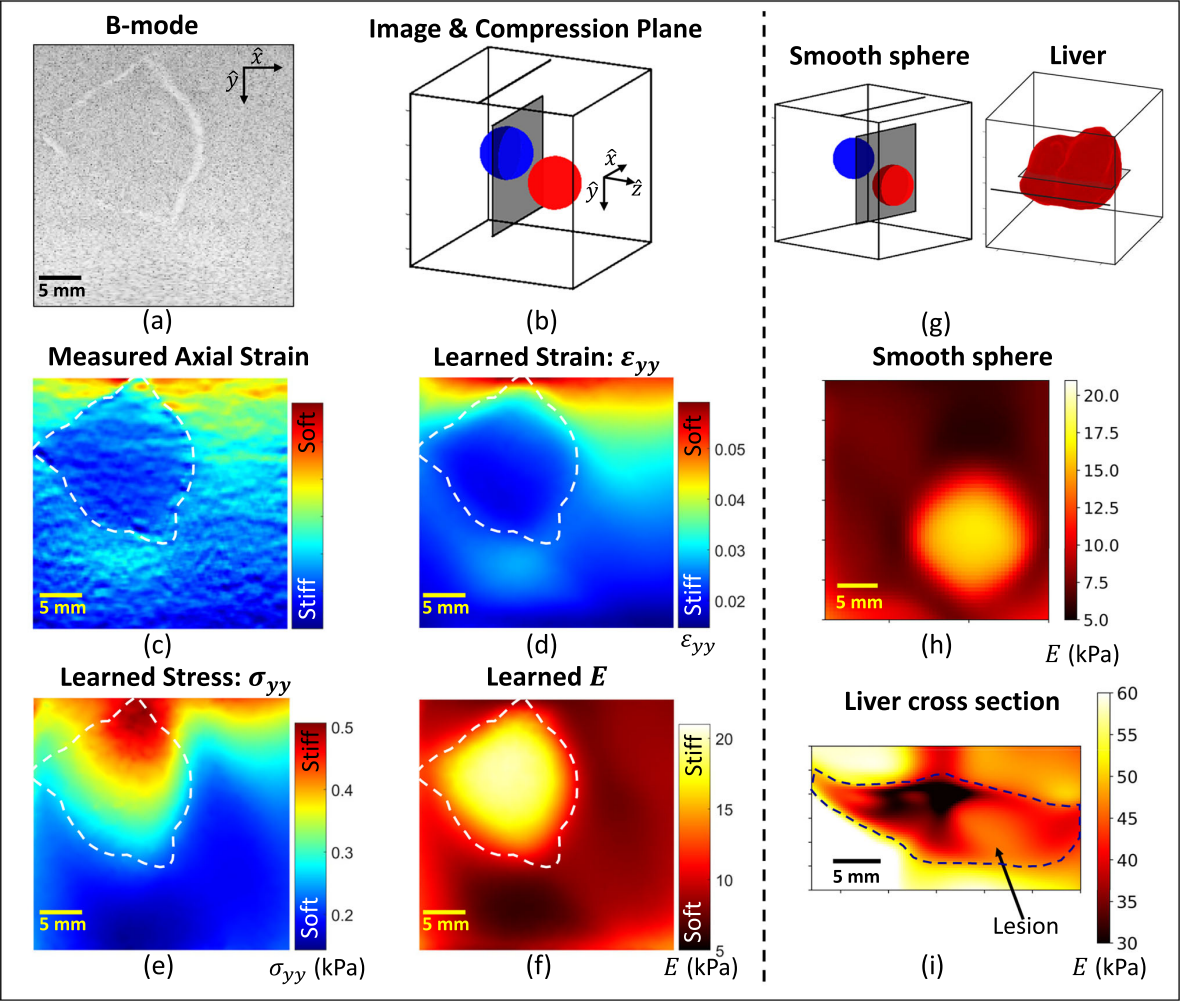
Component data contributing to the formation of a planar elastic modulus image *E* with experimental measurements from one compression plane. The B-mode image (**a**) outlines the inclusion surface in the scan plane, showing spiculations at 12, 5, and 9 o’clock. The compression plane is coincident with the image plane intersecting one of the spheres (**b**). The measured axial strain (**c**) is found from a derivative of the measured displacements, while the learned strain image in (**d**) and the learned stress in (**e**) describe training data components obtained from a fully trained CaNNCM. The Young’s modulus (*E*) image in (**f**) is generated using all Voigt components of stress and strain, not only the axial component shown in (**d**-**e**). Additional single plane *E* images were developed using the compression planes illustrated in (**g**). The *E* image in (**h**) shows the smooth spherical inclusion of Fig. 2a and (**i**) shows a plane through the liver of Fig. 2b, intersecting the left and right lobes, and coincident with the lesion site

### Planar imaging

Measurement data from individual compression planes that were acquired during three separate experiments are displayed in Fig. 3. Each compression plane was used to train a separate CaNNCM through 10 passes. As diagrammed in Figs. 3b and g, each compression was normal to the contact surface and coincident with the elastic modulus image plane. The 8 MHz B-mode image of the two-sphere phantom in Fig. 3a shows the spiky inclusion outline.

Using the RF echo data for the B-mode image, we estimated the strain image in Fig. 3c. It is computed directly from the spatial gradient of axial displacement estimates (*y*-axis). Strain contrast results from a combination of relative material stiffness in the plane and variations in the stress field throughout the volume. Through this cross section of the inclusion, we see noisy strain estimates that provide submillimeter B-mode-like spatial resolution.

The AutoP generated estimates of axial stress and strain computed from $\textrm{FEA}_\sigma$ along the *y*-axis in the same plane as Fig. 3c appear in Figs. 3d and e. These images were obtained from a CaNNCM that was fully trained in 58 min of computation time. The strain images in Figs. 3c and d resemble each other, except for noise and spatial resolution features. Variation in displacement estimates appearing as strain noise in Fig. 3c propagate into the learned strain image of Fig. 3d as a loss of spatial resolution. Combining the six components of stress and strain found once the CaNNCM has converged, we form the planar Young’s modulus image in Fig. 3f.

Planar images of the elastic modulus are accurate within 10%; that is, Fig. 3f reports *E* values near the center of the inclusion to be within 2 kPa of the 20 kPa value obtained independently using materials testing techniques on separate test samples. Similarly, the mean background modulus value is within 0.5 kPa of the 8 kPa value obtained from materials testing.

Figure 3h displays a planar *E* image of the smooth sphere from an adjacent compression plane in the two-sphere phantom. The symmetry of the sphere is clear to see and the accuracy of the modulus image is similar to that of the planar image in Fig. 3f. We also show a plane through the rat liver in Fig. 3i that intersects both lobes at the center of the formalin injection site. The lesion is visible and it presents stiffer than the normal tissue, however, the material properties are overestimated. Image contrast is preserved but elastic modulus accuracy is compromised in planar imaging when the variation in material properties across the volume is large.

**Fig. 4:**
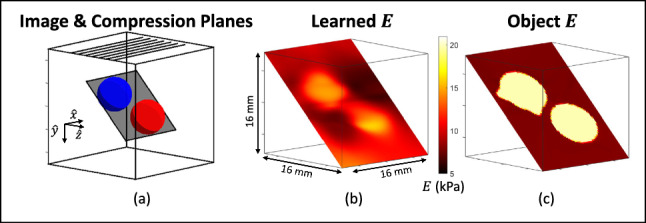
Experimental measurements from the seven compression planes indicated in (**a**) were used to form the volumetric *E* image in (**b**). For reference, the modulus distribution of the object is shown in (**c**) in the same oblique image plane

**Fig. 5:**
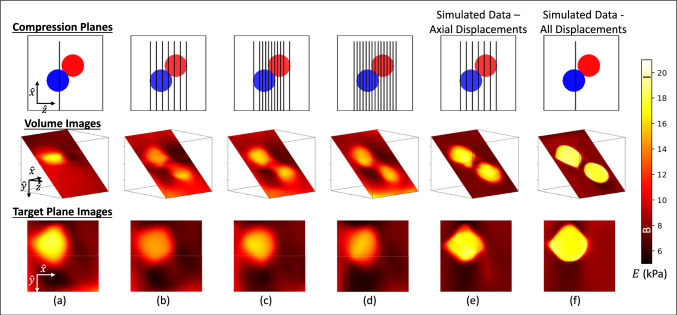
Volumetric imaging of the two-sphere phantom with varying numbers of compression planes. The mean measured *E* intensities from cylindrical test samples are labeled “B" and “I" on the color bar for background and inclusion, respectively

### Volumetric imaging

The learned volumetric elastic modulus image for the two-sphere phantom is displayed in an oblique plane that captures cross sections through both spheres (Figs. 4a and b). This CaNNCM was developed from data acquired in seven compression planes, each in the $x,\,y$ plane of the phantom, as indicated by lines on the top surface in Fig. 4a. The parallel compression planes are separated by 4 mm and the force was applied along the *y* axis. These compression planes are not coincident with the image plane, as they were in Fig. 3.

Modulus image distortions occur when there are errors in force and displacement measurements or when the applied force does not detectably deform all elements of the sampled volume. Both influences reduce the fidelity of the learned *E* image in Fig. 4b, compared to the true elastic-modulus image shown in Fig. 4c. Despite distortions, volumetric imaging of the two-sphere phantom results in *E* inclusion estimates within 5 kPa of the target value, background estimates within 1 kPa of the target value, and a spatial resolution of 6 mm. Spatial resolution was determined by estimating the modulation transfer function (MTF) from the radial edge response of the smooth sphere inclusion. MTF is a unitless real quantity with values ranging from 0 to 1 that describes the efficiency of an imaging technique at transferring Fourier basis functions from the object domain to the image domain (Rossmann 1969). The reported MTF value was measured at the spatial frequency corresponding to MTF=0.2.

The planar images of Fig. 3 produce a higher contrast, higher spatial resolution *E* image that is quantitatively accurate for simple materials, but is biased high in mechanically diverse volumes. Modulus contrast cannot develop in regions that do not deform or in regions where we do not provide displacement measurements. The accuracy of estimating the elastic modulus in a plane is limited in media with high volumetric variation because a single plane of displacements does not sufficiently sample volumetric deformation. Volumetric imaging expands the field of view in which contrast can develop and generates less-biased estimates in all media, but results in a lower image contrast, lower spatial resolution *E* image compared to planar imaging.

The following sections examine data acquisition techniques that improve volumetric image quality. Specifically, we investigate the inclusion of a variety of compression planes to enrich the training environment and speed training while improving volumetric image quality. The main research questions we seek to answer in these sections are (a) with what spacing and orientation should compression planes be provided and (b) can we use the three test statistics to predict when data from additional compression planes enhance or degrade the elastic modulus image.

### Developing volumetric models from parallel planes

Volumetric images require the acquisition of compression planes that span the volume of interest, but what spacing and orientation achieve acceptable quality images in the least amount of training time? Figs. 5a-d display elastic modulus images from four different CaNNCMs that were developed using an increasing number of parallel compression planes. The beam axis remains in the *x*, *y* compression plane for these phantom measurements. The locations of the compression planes are indicated in the first row of Fig. 5 by black lines on the top-surface view of the two-sphere phantom. Results are displayed in the oblique plane for images in the middle row and in the compression plane of Fig. 5a for images in the bottom row, where only the spiculated sphere appears.

The first four columns in Fig. 5 are experimental images developed using, respectively, 1, 7, 11, and 15 compression planes, each separated by 2 or 4 mm. We chose 4 mm spacing as the maximum because hex-elements were spaced 2 mm apart and the inclusion contrast in the volume image of Fig. 5a fades ±2 mm away from the single compression plane. The reduction of modulus intensity as more than one compression plane is added is a consequence of including additional volumes of training data while only providing planes of displacement measurements (Newman et al. 2024b).

The experimental measurements used to form the image of Fig. 5b are simulated and applied to form the image in Fig. 5e. The only difference is that simulated displacements are exact.

The images in Fig. 5f are from a model trained with all three displacement components recorded throughout the entire cubic volume, but generated by applying only one simulated compression plane. These results do not have an experimental analog, but demonstrate that ideal performance is achievable with AutoP, even for a single compression plane, when all force and displacement information is available and accurate. Notice how the spiculated and smooth spheres are easily differentiated in Fig. 5f. Spatial resolution in this modulus image is limited only by the 2 mm FE mesh element size.

**Fig. 6:**
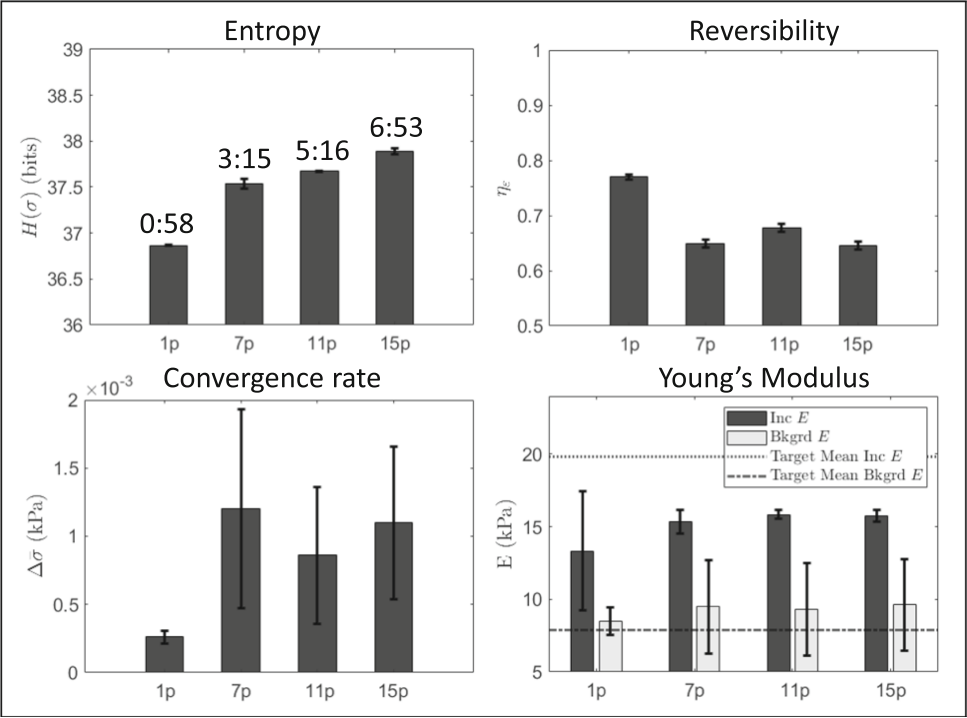
Test statistics measured at the end of training are estimated from training data spanning the volume image region as shown in the middle row of Fig. 5a-d (1 plane (p), 7 planes, 11 planes and 15 planes, respectively). Total computation time during training (hr:min) is displayed above each set of entropy values. The Young’s modulus bar plot reports the mean ± one standard deviation in *E* measured at the center of both inclusions (dark gray) and the background (light gray) for the same three tests. Black dashed lines are the target values from compression testing

Figure 6 examines the three test statistics as well as elastic modulus accuracy and computation time in the four experimental tests described in Fig. 5a-d. Each value is reported from measurements obtained at the end of training. Test statistics labeled entropy, reversibility and convergence rate report the mean value ± one standard deviation across the final three passes of AutoP training.

A single plane exhibits the highest reversibility and the best overall convergence but also the least stress entropy. Adding parallel planes, marginally increases entropy that AutoP struggles to fully assimilate. Figure 6 results motivated the following study exploring changes in the direction of the compression.

**Fig. 7:**
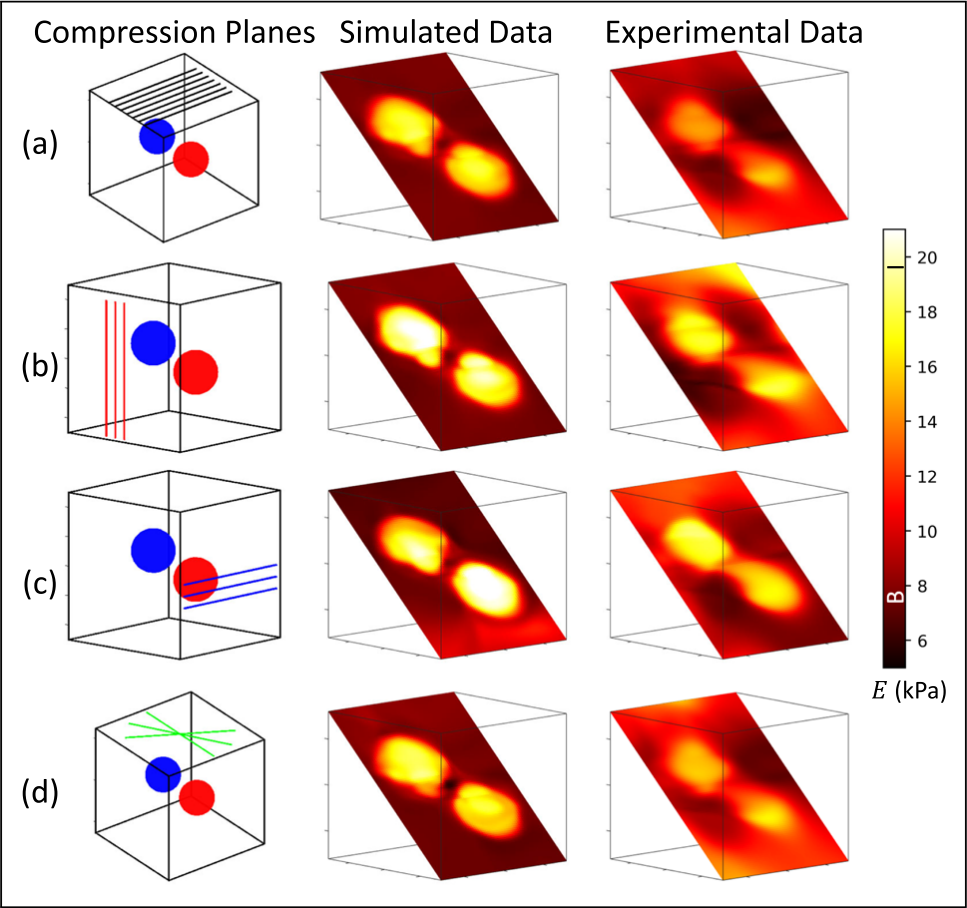
The 7-plane experiments reported in Fig. 5b and 5e for experimental and simulated measurements involving the two-sphere phantom are shown again in the top row (**a**). Beginning with this CaNNCM, we continued training with one set of three additional compression planes indicated in the first column of (**b**)-(**d**)

**Fig. 8:**
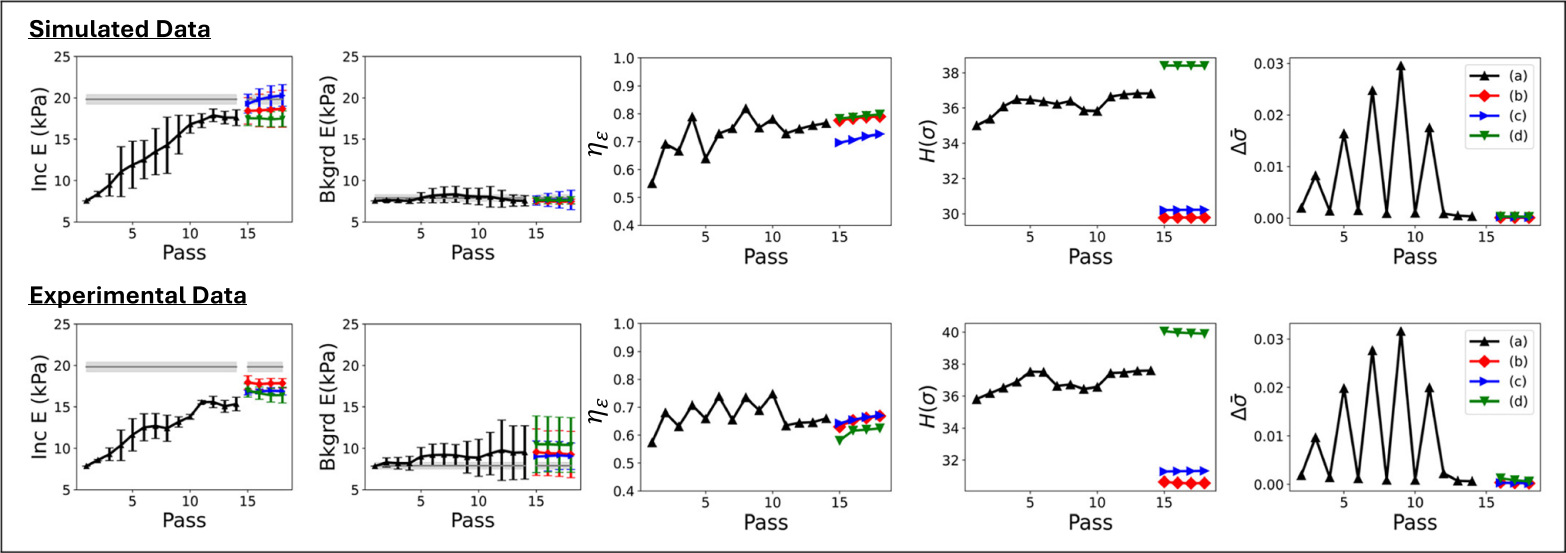
Estimates of the mean *E* values in the inclusion and background regions, volumetric strain reversibility, volumetric stress entropy, and model convergence rate are plotted as a function of training pass. Results for simulated (top row) and experimental (bottom row) phantom measurements are presented. Color relates data points to the compression planes tests described in Fig. 7a-d. The horizontal gray lines in the modulus plots indicate mean ± one standard deviation of the ground truth from independent measurements of the inclusion and background materials

### Continuing training with additional compression planes

The models developed for seven compression planes in Fig. 5b and e appear again in Fig. 7a. From that point, we continued training with three additional compression planes along the directions indicated by the red, blue and green lines in the first column of Fig. 7b-d. Images formed from simulated and experimental force-displacement measurements are shown side by side. Training with three additional compression planes for four passes added 72 min to the overall computation time. We chose the 7-plane results as the starting point as opposed to 11- or 15-planes since we were able to achieve comparable volumetric image quality in the least amount of computation time.

The addition of orthogonal planes in Fig. 7b and c improved the uniformity and image contrast of inclusions in phantom experiments. However, adding the rotated planes along the top of the phantom, as shown in Fig. 7d, produced little change in the image. To help us understand these responses to new data, we present Fig. 8 displaying measurements of the mean inclusion and background *E*, strain reversibility $\eta _{\varepsilon }$, stress entropy $H(\sigma )$, and stress convergence $\Delta \bar{\sigma }$ versus training pass for the results in Fig. 7. The legend in Fig. 8 corresponds to the colors and labels of the three added planes in Fig. 7.

Whereas the results of Fig. 6 are reported at the end of training, Fig. 8 describes the evolution of each test statistic after each training pass. Results for the first 14 passes shown as black points in Fig. 8 include training data developed from only the initial seven parallel compression planes. Large fluctuations between sequential passes are a result of the training schedule that moves from one compression plane to the next. During passes 11-14, all 7-planes were used in training simultaneously. Beginning with pass 15, training resumes with one set of three new compression planes as indicated in Fig. 7. Gaps in the curves of Fig. 8 occur at the transition point. The strain reversibility and stress entropy from pass 15-18 were computed by combining the training data from the end of pass 14 with the newly generated training data. In this way, the two metrics reflect what is *added* to the training environment by including the new compression planes.

In Fig. 8, we find the addition of two orthogonal views (red and blue lines with $\diamond$ and $\triangleright$ markers) improves the accuracy of elastic modulus estimates in the inclusion to nearly 100% as the inclusion intensity increased by a few kPa. Inclusion contrast and accuracy are unchanged after adding the rotated planes (green lines with $\triangledown$ markers). Improvement to the *E* image occurred using the orthogonal planes, as strain reversibly was maintained, stress entropy decreased, and the convergence rate approached zero. No improvement to the *E* image occurred using rotated planes, as strain reversibility was maintained, the stress entropy increased and the convergence rate approached zero.

To interpret the trends in these metrics, recall that AutoP develops an entire volume of stress–strain training data from axial displacement measurements made from each compression plane. As the model is developing, the component of training data that is aligned with the displacement estimates is most accurate; this training data is supported directly by measurements. AutoP is tasked with figuring out the other components of training data at all points throughout the volume from the provided measurements and boundary conditions. In the original compression environment, the most accurate training data are along the axial component within the seven measurement regions. Over many passes, AutoP learned the training data throughout the volume within all six components of stress and strain.

When we provide new measurements aligned with the elevational (blue curves) or the lateral (red curves) directions, the accuracy of the training data improves along those directions. The newly added orthogonal planes align measurements with those components of training data, enhancing the accuracy of that training data. If at the same time, the new training data reinforces the original training data, while maintaining or improving strain reversibility, then the new measurement planes serve only to enhance what has been previously learned with more accurate information. The decrease in stress entropy with maintained strain reversibility indicates this reinforcement since we combine the new training data with the training data from the original set. Nothing novel was added to the training environment, but the additional measurement information could be integrated into the CaNNCM while improving the accuracy in a new component of training data.

**Fig. 9:**
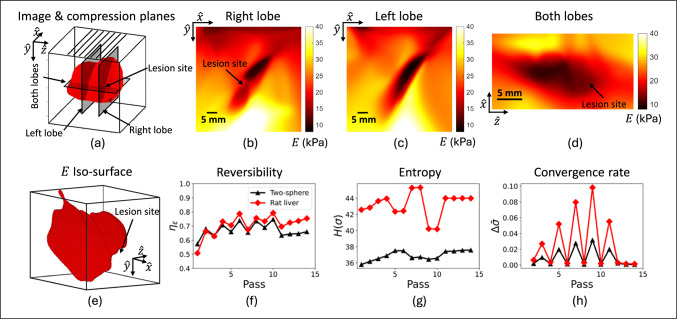
Volume images and test statistics as a function of training pass for the rat liver. The seven compression planes used in training are illustrated in (**a**), along with the three image planes used to display the *E* images of (**b**)-(**d**). Dark regions in the images correspond to the soft, normal liver tissue. An iso-surface rendering of the normal liver tissue was generated from the 3D *E* image in (**e**). Reversibility, entropy and convergence rate are shown for the rat liver test and corresponding two-sphere test in (**f**)-(**h**)

**Table 1:** Estimates of component *E* (mean ± standard deviation) for the rat liver

Component	Measured *E* (kPa)	Planar Image *E* (kPa)	Volume Image *E* (kPa)
Normal Liver Tissue	$4.7 \pm 1.3$	$31.4\pm 0.7$	$10.8\pm 0.4$
Lesion Site	$14.3 \pm 5.6$	$45.8\pm 0.4$	$17.7\pm 0.6$
Background Gelatin	$17.4 \pm 1.4$	$57.7\pm 0.9$	$27.3\pm 0.9$

Measured values are from compression testing on cubic samples after scanning. Refer to Fig. 3i for the planar image and Fig. 9d for the volume image

The rotated planes (green curves) increased the total stress entropy while providing measurements in the same direction as the original compression planes. The increased entropy signals that novel training information was added to the learning environment, but the maintained strain reversibility indicates that the new information has not been successfully transferred to the CaNNCM. An increase to stress entropy must be accompanied by an increase to strain reversibility if it is to aid in model development. Because the CaNNCM could not adapt to the added information with the provided rotated planes, the model mostly maintained.

### Rat liver imaging

We also examined volumetric image formation in the *ex vivo* rat liver. Our goal in this section was to demonstrate the ability of AutoP to image media with variations in material properties greater than the two-sphere phantom. Elastic modulus images of the liver volume through three image planes are shown in Fig. 9. The images were formed by training a CaNNCM using seven parallel compression planes spaced 4 mm apart.

Figure 9a identifies the locations of seven compression planes used for training and the three image planes displayed in Fig. 9b-d. The surface rendering in Fig. 9a was generated using a 3D scanner app on an apple iPhone 13, not using AutoP. The surface rendering of Fig. 9e represents the normal liver tissue volume as an iso-surface, treating *E* values less than the target lesion *E* (14.3 kPa) as normal tissue. The lesion region appears as a deficit in the iso-surface.

**Fig. 10:**
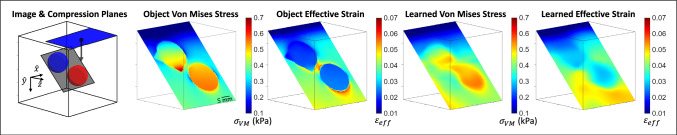
Results of retrospective numerical experiments using alternative compression patterns. Images labeled “object" are formed using standard FEA modeling with known geometry and material properties as reported in Fig. 4c. Images labeled “learned" use the CaNNCM-defined material properties reported in Fig. 7c

The right-lobe image of Fig. 9b and the two-lobe image of Fig. 9d are in planes that intersect the formalin lesion. The lesion appears stiffer (brighter) than the surrounding liver tissue, but softer (darker) than the gelatin surrounding the liver. Each region is discernible in the elastic modulus images, although the $\sim$6 mm spatial resolution hinders our ability to clearly map the lesion shape. The sharp boundary of the planar image shown in Fig. 3i is blurred in the volumetric image shown in Fig. 9d. The quantitative estimation of *E* in the volume images of Fig. 9 are overestimated but in much closer agreement to the measured values from compression testing than the planar image of Fig. 3i. Table 1 compares quantitative *E* estimates in the rat liver components between planar and volumetric images in the same image plane.

Figure 9f-h compare the reversibility, entropy and convergence rate, respectively, between the two-sphere phantom and the rat liver. In both cases, seven compression planes spaced 4 mm apart were applied during training. Using the same amount of compression (3% total strain) and spanning the same total volume, the two curves provide a comparison of how the dynamic range of the volumetric material properties influences training. A comparatively larger liver volume with a stiffness that varies from the background more than the sphere stiffness varied from its background resulted in greater stress entropy in the training data and greater reversibility in the liver model than the two-sphere model. Based on the test statistics, we find that AutoP was able to make use of the greater information content in the liver phantom when developing that CaNNCM.

### Applying the learned material properties to image stress and strain

A unique feature of the AutoP approach to elasticity imaging is its ability to accurately model stress and strain throughout a compressed volume. Once a CaNNCM is trained, numerical experiments can be performed on the meshed volume to predict the stress–strain patterns occurring under different experimental conditions.

Figure 10 is the result of combining FEA and the CaNNCM developed for the images in Fig. 7c to predict the stress and patterns in the same oblique plane after the compressor was enlarged to cover half of the top phantom surface. In this way, the consequences of modifying the loading patterns and other boundary conditions can be explored numerically.

## Discussion

The results of Figs. 5,6, 7 and 8 help us understand how AutoP learns to model stress and strain in a volume as the software is exposed to various compression plane measurements. We saw that adding more compression planes always increases training time but does not always improve image quality or modulus estimation accuracy. So, what strategy should we adopt to encourage efficient learning?

We note that measurements from distinct compression planes each form an entire volume of training data (Fig. 1b). While each compression plane adds new information about materials in the volume, it also adds force-displacement errors that introduce inconsistencies in the training data volumes. The learned training data are said to be consistent when the networks drive results from different compression planes toward the same material properties. Networks trying to converge on inconsistent training data will seek a compromised solution that tends to flatten the field of elastic modulus estimates.

We found that training with one compression plane to form an elastic modulus image results in the greatest stress convergence with the highest spatial resolution and image contrast. Figure 6 shows that training with a single compression plane generates the least stress entropy but the most reversibility. The contrast and resolution for the planar image in Fig. 5a are superior to the planar image from multi-plane training (Figs. 5b-d). The reason is that inconsistencies cannot arise using one compression plane, so the model for the image in Fig. 5a converges quickly and with much detail. However, the lack of direct information about the medium outside of the compression plane leads to inaccurate modulus estimates throughout the volume. The test statistics inform us that AutoP requires additional information to further narrow the range of potential solutions.

Figure 5 results show that we must train with measurements acquired throughout a volume that spans the internal heterogeneities when the clinical objective extends beyond planar-image contrast generation and toward achieving accurate estimates of the elastic modulus throughout the volume. The price paid for improving accuracy is greater training time and a loss of image contrast and spatial resolution compared to planar imaging. This loss of image quality is due to inconsistencies in training data from measurement errors.

Monitoring the statistical measures of Figs. 6 and 8 provides clues about the loss. Additional stress entropy was generated by adding parallel planes (Fig. 6), but AutoP struggled to capture that increased entropy as strain reversibility was seen to change minimally. Each new parallel measurement plane increased entropy, but the greater information capacity may not translate into a consistent training message. AutoP responds to inconsistent training data by adjusting network weights to keep values closer to the spatially averaged modulus value learned by the MPN. When the compression planes are without measurement errors, as occurred with simulated measurements in Fig. 5e, we found similar deformation patterns from each volume of training data that resulted in high strain reversibility and rapid model convergence.

Adding three orthogonal planes to the seven original planes (Figs. 7b and c) narrows the stress bandwidth, which lowers stress entropy. These changes indicate that the additional information reinforces the training data learned previously, and it leads to minor improvements in E image quality. Adding three rotated planes to the seven original planes (Fig. 7d) broadens the bandwidth for stress to increase stress entropy, but that information was not transferred into the deformation model as strain reversibility did not increase and image quality did not improve. We found that increases in entropy must be supported by greater strain reversibility to indicate there is a consistent training message.

In all cases, additional measurements had only a minor influence on the *E* images, meaning most of model development occurred during the original stages of training. Adding the data from three compression planes to training, as shown in Fig. 7, provided little or no improvement to image quality. The most accurate models are found when accurate displacement measurements span the sample volume. The value of the three test statistics to the learning process is not in evaluating the quality of the model, but in guiding decisions about when additional data may be beneficial and when to stop training.

Our findings suggest a strategy for imaging the elastic modulus of tissues using AutoP with measurements from US linear arrays. (a) Acquire parallel compression planes separated by the elevational resolution of the probe over the entire volume. (b) Train on single planes of interest to generate qualitative planar elastograms with high image quality. (c) Train on multiple compression plane data to improve the accuracy of the elastic modulus estimates and form images in any arbitrary plane throughout the volume. (d) Use the three test statistics to decide when to stop training.

This procedure helped us decide to train using parallel compression planes separated by the elevational beamwidth for the rat liver in Fig. 9. We found that large variations in medium properties enhanced the learning environment as those heterogeneities resulted in a high stress entropy, strain reversibility above 0.7, and a low convergence rate, indicating that the supplied compression data was sufficient. The corresponding modulus estimates within the normal liver and formalin-induced lesion site were within 3-5 kPa of the independently measured values.

Experimental measurement will always introduce inconsistencies into training. Force errors are usually minimal and determined by noise in force sensors. Displacement errors from the GLUE speckle-tracking algorithm applied to linear array US measurements are more complex (Hashemi and Rivaz 2017). GLUE estimates local displacements in a scan plane by analyzing echo data at multiple scales. Displacement error results from reduced inter-frame echo coherence between sequential scans recorded as the medium is compressed. Echo decorrelation occurs in low echo SNR recording conditions or when scatterers move out of the scan plane during compression (Ashikuzzaman et al. 2024). We previously found that displacement errors appear as spatially uniform bias rather than variance.

The AutoP training process only depends on the imaging modality for displacement estimates. The technique itself is modality independent. While the GLUE algorithm is well suited to the needs of AutoP training using ultrasound data from linear arrays, techniques that provide three components of displacement everywhere in the volume for each applied load step could further improve training data consistency during multi-plane training (Fig. 5f).

Other factors such as the surface geometry and boundary conditions can impact the training environment. We chose to embed the liver in a gelatin cube to simplify and standardize the cubic surface geometry. This let us compare training outcomes between test objects with different internal material properties while using the same training procedure. The training strategy varies somewhat with the shape of the medium and the imaging device used to track displacements. However, the three test statistics guided data acquisition to form images targeting specific imaging objectives. For example, focal lesion detection tasks may only require elastic modulus contrast (Sigrist et al. 2017; Sandulescu et al. 2012), whereas monitoring tumor progression and response to therapy likely benefits from consistent and accurate estimation of 3D properties (Fernandes et al. 2019; Nabavizadeh et al. 2018).

It is also important to uncover accurate 3D tissue material properties when the objective is to study the responses of tissues to applied forces. CAD-based assisted surgical planning (Yang et al. 2025) and computation modeling of tissues (Sel et al. 2024) are two approaches that actively aim to quantify tissue mechanical responses to applied stimuli for predictive modeling. A limitation of these approaches is that individual patient tissue material properties are unknown and must be assumed. In Fig. 10, we demonstrated that a trained CaNNCM can be used to image the stress and strain responses under arbitrary loading conditions. The mechanical response can be captured by performing numeric simulations with learned material properties, measured surface geometry, and user-defined boundary conditions.

## Summary

Accurate 3D material property distributions can be formed using quasi-static compression data from linear array transducers. AutoP is fundamentally a method of uncovering the heterogeneous stress and strain fields in 3D. Knowledge of the quantitative stress and strain enables accurate estimates of the elastic modulus, which can be used to simulate the mechanical responses of tissues under arbitrary loading conditions. The limited planar measurement data that is available for training is well suited to data-driven inversion techniques. We have outlined a general procedure for imaging with linear array US transducers that results in 3D elastic-modulus images with 6 mm spatial resolution (at 8 MHz) and estimates within a few kPa of the target values in 3 h of training time. We learned that accessible measurement information comes from measurements that contribute to consistent training data. Our test statistics helped us to understand if measurements efficiently informed the construction of a constitutive model using neural networks.

## Data Availability

The data presented in this article are publicly available at zenodo.org/records/17095486
